# In vivo isolated kidney perfusion with tumour necrosis factor **α** (TNF-**α**) in tumour-bearing rats

**DOI:** 10.1038/sj.bjc.6690067

**Published:** 1999-02

**Authors:** A H van der Veen, A L B Seynhaeve, J Breurs, P T G A Nooijen, R L Marquet, A M M Eggermont

**Affiliations:** Department of Surgery, University Hospital Rotterdam Dijkzigt, Daniel den Hoed Cancer Centre, Rotterdam, The Netherlands; Department of Pathology, University Hospital Nijmegen, Nijmegen, The Netherlands

**Keywords:** tumour necrosis factor α, isolated kidney perfusion, rats

## Abstract

Isolated perfusion of the extremities with high-dose tumour necrosis factor α (TNF-α) plus melphalan leads to dramatic tumour response in patients with irresectable soft tissue sarcoma or multiple melanoma in transit metastases. We developed in vivo isolated organ perfusion models to determine whether similar tumour responses in solid organ tumours can be obtained with this regimen. Here, we describe the technique of isolated kidney perfusion. We studied the feasibility of a perfusion with TNF-α and assessed its anti-tumour effects in tumour models differing in tumour vasculature. The maximal tolerated dose (MTD) proved to be only 1 μg TNF-α. Higher doses appeared to induce renal failure and a secondary cytokine release with fatal respiratory and septic shock-like symptoms. In vitro, the combination of TNF-α and melphalan did not result in a synergistic growth-inhibiting effect on CC 531 colon adenocarcinoma cells, whereas an additive effect was observed on osteosarcoma ROS-1 cells. In vivo isolated kidney perfusion, with TNF-α alone or in combination with melphalan, did not result in a significant anti-tumour response in either tumour model in a subrenal capsule assay. We conclude that, because of the susceptibility of the kidney to perfusion with TNF-α, the minimal threshold concentration of TNF-α to exert its anti-tumour effects was not reached. The applicability of TNF-α in isolated kidney perfusion for human tumours seems, therefore, questionable. © 1999 Cancer Research Campaign


*British Journal of Cancer* (1999) **79**(3/4), 433–439
© 1999 Cancer Research Campaign
Article no. bjoc.1998.0067

### In vivo isolated kidney perfusion with tumour necrosis factor α (TNF-α) in tumour-bearing rats

**AH van der Veen[1], ALB Seynhaeve[1], J Breurs[1], PTGA Nooijen[2], RL Marquet[1] and AMM Eggermont[1]**

[1]Department of Surgery, University Hospital Rotterdam Dijkzigt, Daniel den Hoed Cancer Centre, Rotterdam, The Netherlands; [2]Department of Pathology, University Hospital Nijmegen, Nijmegen, The Netherlands

**Summary** Isolated perfusion of the extremities with high-dose tumour necrosis factor α (TNF-α) plus melphalan leads to dramatic tumour response in patients with irresectable soft tissue sarcoma or multiple melanoma in transit metastases. We developed in vivo isolated organ perfusion models to determine whether similar tumour responses in solid organ tumours can be obtained with this regimen. Here, we describe the technique of isolated kidney perfusion. We studied the feasibility of a perfusion with TNF-α and assessed its anti-tumour effects in tumour models differing in tumour vasculature. The maximal tolerated dose (MTD) proved to be only 1 μg TNF-α. Higher doses appeared to induce renal failure and a secondary cytokine release with fatal respiratory and septic shock-like symptoms. In vitro, the combination of TNF-α and melphalan did not result in a synergistic growth-inhibiting effect on CC 531 colon adenocarcinoma cells, whereas an additive effect was observed on osteosarcoma ROS-1 cells. In vivo isolated kidney perfusion, with TNF-α alone or in combination with melphalan, did not result in a significant anti-tumour response in either tumour model in a subrenal capsule assay. We conclude that, because of the susceptibility of the kidney to perfusion with TNF-α, the minimal threshold concentration of TNF-α to exert its anti-tumour effects was not reached. The applicability of TNF-α in isolated kidney perfusion for human tumours seems, therefore, questionable.

**Keywords:** tumour necrosis factor α; isolated kidney perfusion; rats

Tumour necrosis factor α (TNF-α) in combination with melphalan with or without the addition of γ-interferon is presently used in the treatment of patients with ‘in transit’ metastases of malignant melanoma and patients with locally advanced soft tissue sarcoma (STS). In both groups of patients, the cytokine TNF-α and the cytotoxic agent melphalan are used in isolated perfusion of the limb (ILP). The efficacy in the treatment of locally advanced soft tissue sarcomas, characterized by high response rates (>80%) resulting in limb salvage in about 80% of the patients, has now been well established by published reports of multicentre experiences in up to 200 perfusions (Eggermont et al, 1993, 1996*a*, 1996*b*). Initially, the procedure was developed in the more traditional field of applying ILP, for example in the treatment of in transit melanoma metastases, and high complete remission rates in melanoma patients have been reported (Liénard et al, 1992, 1994).

The exact mechanism of TNF-α anti-tumour activity has not yet been fully elucidated, but a number of theories exist (Sidhu and Bollon, 1993). TNF-α has direct and indirect effects. It can induce tumour-specific immunity (Spriggs and Yates, 1992) and is cytotoxic/cytostatic for some tumour cell lines in vitro (Dealtry et al, 1987). Its direct effect on tumour cells was proven shortly after the discovery of the cytokine (Watanabe et al, 1988), but the indirect effects probably play a more important role. The detrimental effects on the tumour-associated vasculature is mediated by endothelial cells (Shimomura, 1988), however, the effect on the microvasculature seems to be dose dependent (Fajardo et al, 1992).

In contrast, high dosages of TNF-α exert a number of undesirable effects. The maximum tolerated dose (MTD) in humans is 350 μg m$^{-2}$ (Brouckart et al, 1986; Asher et al, 1987) which is 10–50 times lower than the desired anti-tumour dose when given intravenously. Because of severe toxicity, observed already at relatively low doses of TNF-α after systemic administration, in phase I/II clinical trials, virtually no tumour responses were observed (Feinberg et al, 1988; Spriggs et al, 1988). This is not surprising because TNF-α was never administered systemically at doses that might have anti-tumour activity. This led Lejeune et al (1993) to the development of the isolated perfusion with TNF-α together with melphalan and γ-interferon. The successful application of TNF-α in this setting warrants that the applicability of TNF-α in isolated organ perfusion setting is investigated. Isolated single-lung perfusion with TNF-α proved to be safe (Pogrebniak et al, 1994; Weksler et al, 1994; Pass et al, 1996). Reports on the use of TNF-α in isolated liver perfusion at the National Cancer Institute in the USA (Fraker et al, 1994) as well as by our group (Borel Rinkes et al, 1997) have appeared very recently.

Here, we report on the development of in vivo isolated kidney perfusion and its anti-tumour effects in two tumour models.

*Received 10 February 1998*
*Revised 14 May 1998*
*Accepted 21 May 1998*

*Correspondence to:* AH van der Veen, Department of Surgery, University Hospital Rotterdam Dijkzigt, Rotterdam, The Netherlands, PO Box 2040, 3000 CA Rotterdam, the Netherlands

### MATERIALS AND METHODS

### Animals

Male rats of the inbred WAG-Rij strain (Harlan CPB, Austerlitz, The Netherlands), weighing 250–300 g were used. Rats were kept under standard laboratory conditions. All rats were fed a standard laboratory diet (Hope Farms, Woerden, the Netherlands). The

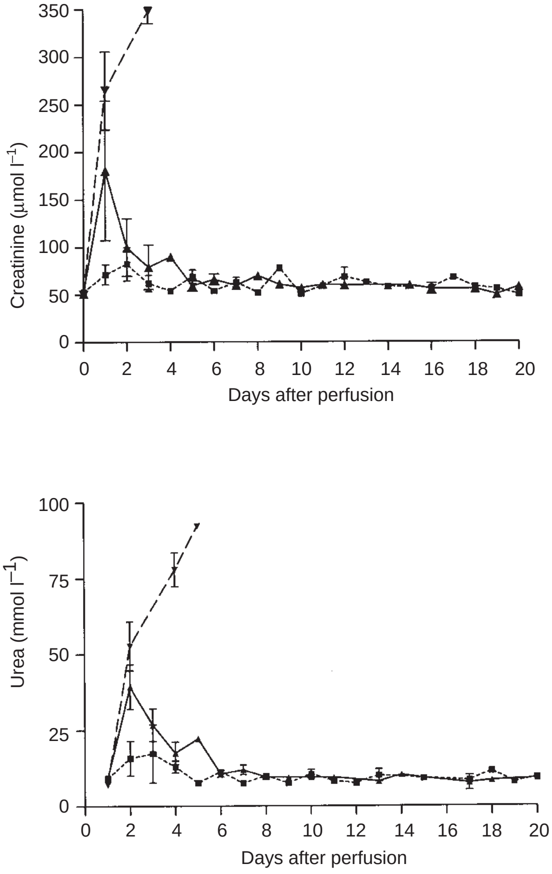

**Figure 1** Course of kidney function parameters as a function of time (days) after isolated kidney perfusion with 1 μg TNF-α in a roller-pump regulated perfusion setting. After 15 min perfusion, urea and creatinine levels returned to normal in sham (■) and 1 μg TNF-α groups (▲), after 2 μg TNF-α (▼) rats were sacrificed after 2–3 days in bad clinical condition (values depicted are a mean of six rats ± s.d.)

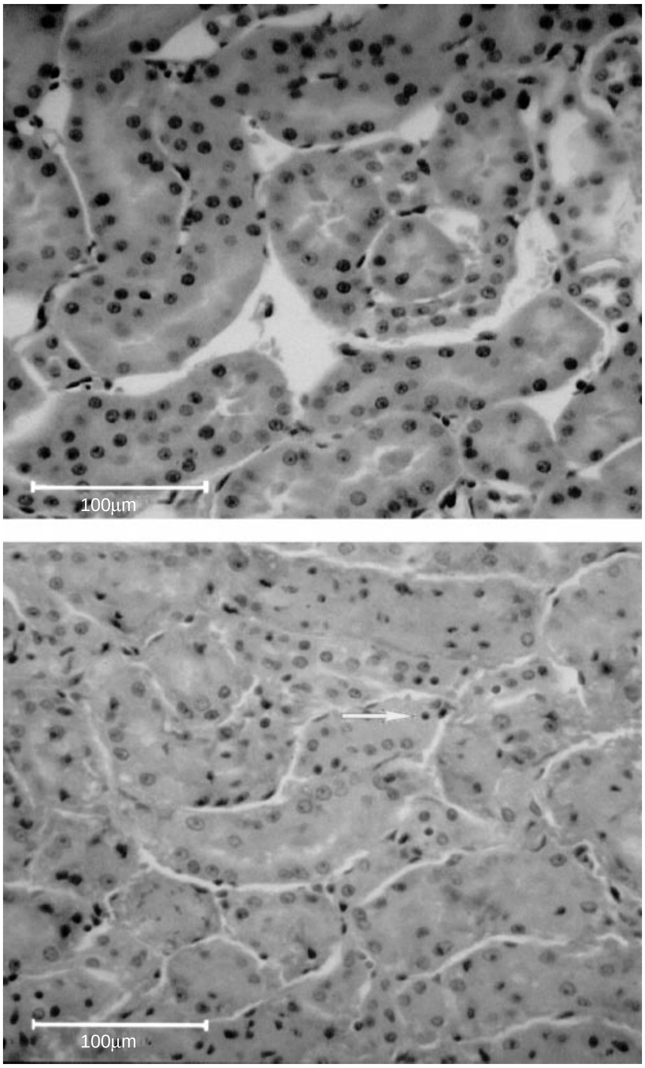

**Figure 2** (**A**) Haematoxylin and eosin (HE) stained section (125 ×) of a kidney 24 h after perfusion with perfusate only. See text for explanation. (**B**) HE-stained section of a kidney 24 h after 15-min perfusion with 2 μg TNF-α. Focal tubular necrosis is indicated (arrow)

experimental protocols adhered to the rules laid down in the ‘Dutch Animal Experimentation Act’ (1977) and the published ‘Guidelines on the Protection of Experimental Animals’ by the council of the EC (1986). The specific protocol was approved by the ‘Committee on Animal Research’ of the Erasmus University Rotterdam, The Netherlands.

### Tumour necrosis factor α

Recombinant human TNF-α (rhTNF-α) was kindly provided by Boehringer Ingelheim, Ingelheim, Rhein, Germany. rhTNF-α had a specific activity of $5.8 \times 10^7$ U $mg^{-1}$ as determined in the murine L–M cell assay (Kramer and Carver, 1986). Endotoxin levels were less than 1.25 endotoxin U $mg^{-1}$ protein. TNF-α was delivered in 0.5-ml vials in a concentration of 2.4 mg $ml^{-1}$.

### Melphalan

Melphalan (Alkeran, 50 mg per vial, Wellcome, Beckenham, UK) was diluted in 10 ml of diluent solvent. Further dilutions were made in 0.9% sodium chloride to give a volume of 0.5 ml in the perfusion circuit.

### Unilateral nephrectomy

It is probable that when two kidneys are perfused in vivo, kidney function as measured by blood urea nitrogen (BUN), creatinine and electrolytes remains stable after isolation and perfusion of one kidney. To analyse to what extent renal function is compromised by unilateral nephrectomy, five rats underwent a unilateral nephrectomy and at regular intervals BUN, creatinine and electrolytes were recorded. The right kidney was chosen for anatomic reasons. The left kidney shows compensatory hypertrophy: the size of the kidney is larger, there is a gain in cell volume and diameter of the glomerulus as well as a larger volume of the tubule (Fine, 1986).

Operative procedures were carried out under clean conditions. Anaesthesia was induced with ether (diethylether p.a. Merck, Darmstadt, Germany). The abdomen was shaved and prepped with ethanol 70%. Through a lumbotomy, the right kidney was exposed and freed from its surrounding fat. Ureter, renal artery and vein were dissected and tied with 4-0 silk sutures (B Braun, Melsungen,

 *© Cancer Research Campaign 1999*

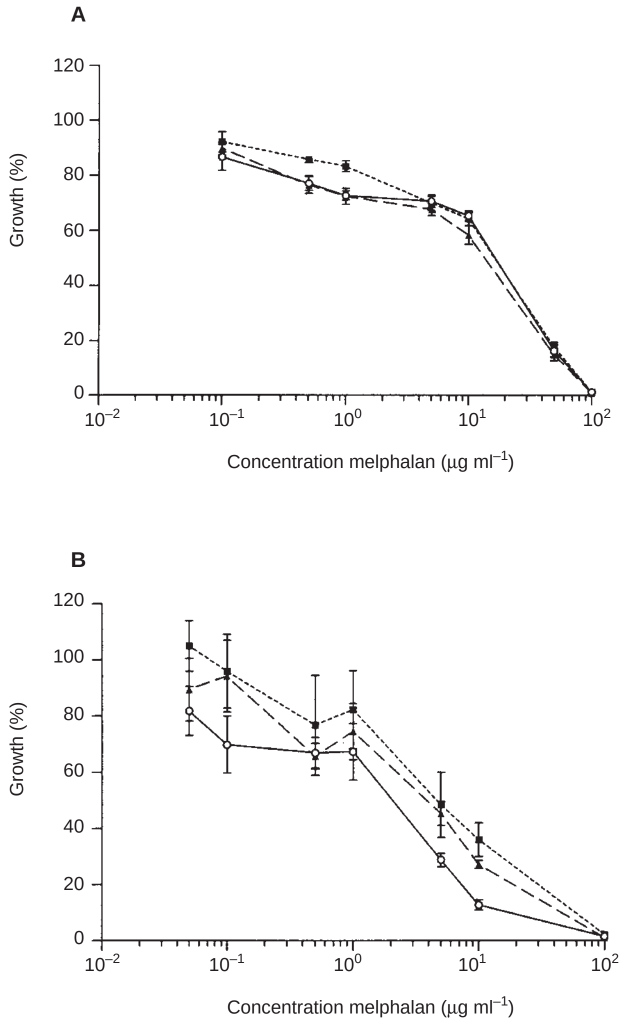

**Figure 3** (**A**) Dose–response curve of CC531 colon adenocarcinoma to melphalan in the absence or presence of various concentrations TNF-α, as determined in the sulphorhodamine B assay (■, 0 µg ml$^{-1}$ TNF-α; ▲, 0.5 µg ml$^{-1}$ TNF-α; ○, 10 µg ml$^{-1}$ TNF-α; for clarity concentrations between 0.5 and 10 µg ml$^{-1}$ TNF have been omitted). (**B**) Dose–response curve of ROS-1 osteosarcoma to melphalan in the absence or presence of various concentration TNF-α, determined in the sulphorhodamine B assay (■, 0 µg ml$^{-1}$ TNF-α; ▲, 0.5 µg ml$^{-1}$ TNF-α; ○, 10 µg ml$^{-1}$ TNF-α)

Germany). The right kidney was removed and the lumbotomy closed with 2-0 silk in a running way. After 3 weeks, the animals were sacrificed.

### Perfusion fluid

In our experiments, a modified Krebs–Henseleit solution (sodium chloride 118.00 mmol l$^{-1}$, calcium chloride 2.52 mmol l$^{-1}$, magnesium sulphate 1.66 mmol l$^{-1}$, sodium bicarbonate 24.88 mmol l$^{-1}$, potassium dihydrogen phosphate 1.18 mmol l$^{-1}$ + D-glucose 5.55 mmol l$^{-1}$, sodium pyruvate 2.00 mmol l$^{-1}$, mannitol 33.00 mmol l$^{-1}$) was used as perfusate.

### Operative technique: isolated kidney perfusion (IKP)

Anaesthesia was induced with ether. A Zeiss operative microscope (Carl Zeiss, Germany) was used. The abdomen was shaved and prepped with ethanol 70%. Through a median laparotomy, the left kidney and vessels were exposed in the retroperitoneum by sharp and blunt dissection. Branches of the renal artery and vein (adrenal and spermatic) were dissected when needed and temporarily occluded with ligatures. A tobacco-pouch ligature was placed in the vein with Nylon 9–0 (SSC, B Braun). A bolus of 50 IE heparin (Heparin Leo, Weesp, the Netherlands) was injected intravenously. Isolation of the renal artery and vein was carried out by means of microvessel clamps. Via a venotomy and arteriotomy, the vessels were cannulated with Silastic tubing (0.025 in. ID, 0.047 in. OD and 0.012 in. ID, 0.025 in OD respectively, Dow Corning, Michigan, USA). Another bolus of 50 IE heparin was added to the perfusion circuit.

### *Perfusion model*

Isolated kidney perfusion was performed to design a model resembling isolated limb perfusion in which TNF-α is administered to the oxygenated extracorporeal perfusion circuit. In this model, 1, 2 or 5 µg TNF-α was added to the perfusion circuit. Perfusion with TNF-α was carried out for 15 min. Thus, TNF-α was allowed to pass the kidney multiple times.

Flow through the kidney was regulated by a non-pulsatile roller pump (Watson-Marlow 505U, Falmouth, UK). Perfusion pressure was recorded on a Datex AS/3 monitor and kept between 100 and 120 mmHg, adjusting the flow generated by the roller pump accordingly. Perfusion fluid was warmed to approximately 37°C by countercurrent (Polyscience 210, Merck, Amsterdam, the Netherlands). Temperature was recorded (Thermodig KJ-11, Mera, Benelux).

Flow through the kidney was approximately 1 ml min$^{-1}$. The reservoir was gassed with carbogen (95% oxygen, 5% carbon dioxide gas mixture), to keep the oxygen pressure of the perfusate at 350–400 mmHg and the saturation at 99.5%.

When TNF-α and/or melphalan was added to the perfusion circuit, a washout was carried out with 6 ml perfusion fluid (about four times the intravascular kidney volume).

At the end of the perfusion period, the venotomy was closed by tightening the tobacco-pouch ligature. Arteriotomy was closed with Nylon 10–0 (SSC, B Braun). The laparotomy was closed with silk 2-0 (B Braun) in one layer in a running way. Total operation time varied between 90 and 120 min. Blood loss was kept to a minimum.

During the recovery period, the animal was kept warm with a 200-W lamp and then returned to his cage.

### Parameters and histology

Weight loss was recorded after operation. In the post-operative period, the animals were observed at regular intervals for signs of toxicity, and deaths were recorded. Clinical condition (skin, eyes, stools and behaviour) was judged. Renal function was assessed through blood urea nitrogen and creatinine levels as well as electrolytes in plasma.

For histopathological analysis, two rats for each TNF-α concentration were sacrificed 24 h after treatment. Kidneys were fixed (emersion method) in 4% formaldehyde solution and embedded in paraffin. Care was taken to keep the time of perfusion fixed, the warm ischaemia period as short as possible, and time between nephrectomy and fixation constant. Sections of the kidneys were stained with haematoxylin and eosin.

© Cancer Research Campaign 1999

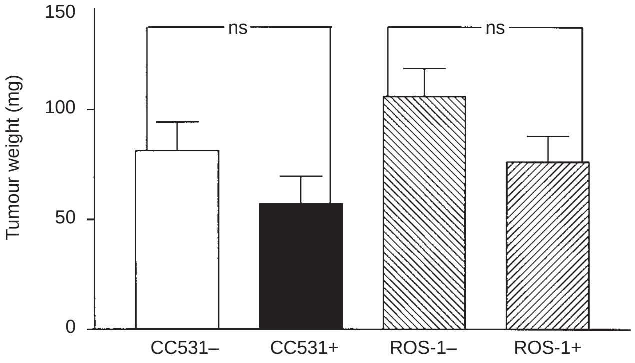

**Figure 4** Growth inhibition of CC 531 adenocarcinoma and ROS-1 osteosarcoma in the subrenal capsule assay after isolated kidney perfusion with 1 μg TNF-α combined with 500 μg melphalan (CC531–, sham; CC531+, treated; ROS-1–, sham; ROS-1+, treated; data shown are mean values of nine rats ± s.d. *P* = n.s.)

For analysis of the vasculature of the tumours, the same procedure was followed.

### Tumour models

#### *Colon carcinoma CC 531*

The 1,2-dimethylhydrazine-induced, moderately differentiated colon adenocarcinoma CC 531 (Marquet et al, 1984), transplantable in syngeneic Wag-Rij rats, was used. The tumour is weakly immunogenic, as determined by the immunization-challenge method of Prehn and Main (1957). The tumour was maintained in vitro in RPMI-1640 medium supplemented with 5% fetal calf serum (virus and mycoplasma screened), 1% penicillin (5000 U $ml^{-1}$), 1% streptomycin (5000 U $ml^{-1}$) and 1% L-glutamine (200 mM). All supplementaries were obtained from Gibco (UK). Before usage, the cells were trypsinized (5 min, 37°C), centrifuged (5 min, 700 ***g***), resuspended in RPMI-1640 and counted. Viability was measured with trypan blue (0.3% in a 0.9% sodium chloride solution). Viability always exceeded 95%.

For in vivo studies, the tumour was inoculated in the flank of syngeneic Wag-Rij rats, where it was allowed to grow until the time of the experiment.

#### *Osteosarcoma ROS-1*

The ROS-1 osteosarcoma (transplantable to Wag-Rij rats) was used in the second series of experiments. This tumour originated spontaneously in the tibia of a rat. The ROS-1 cells grow as a monolayer in Dulbecco's modified Eagle medium. To this medium, 5% fetal calf serum and glutamic acid (Gibco, Paisley, UK) was added. Cells were maintained in a humidified atmosphere of carbon dioxide/air (5/95) at 37°C. From the tissue cultures, new solid tumours were produced by serial inoculation in the flank, where it was allowed to grow until the time of the experiment.

### Experimental design

For in vitro testing of tumour cell lines for susceptibility to TNF-α and melphalan, tumour cells were seeded at $1 \times 10^4$ cells per well in flat-bottomed 96-well microtitre plates (Costar, USA) in a final volume of 0.2 ml medium per well, and incubated at 37°C in 5% carbon dioxide for 48 h in the presence of different concentrations of rhTNF-α and melphalan. Concentrations of TNF-α used were 0.001, 0.01, 0.1, 1, 10 and 100 μmol $ml^{-1}$. Concentrations of melphalan used were 0.04, 0.1, 0.9, 5 and 8 μmol $ml^{-1}$.

Growth of tumour cells was measured using the sulphorhodamine-B assay according to the method of Skehan et al (1990). Eight replicate experiments were performed. Tumour growth was calculated using the formula: tumour growth = (test well/control) × 100 per cent. The drug concentration reducing the absorbance to 50% of control ($IC_{50}$) was determined from the growth curves.

For the in vivo isolated kidney perfusion (in vivo tumour model) the subrenal capsule assay (SRCA) was used. Recipient animals were anaesthetized with ether, a midline incision was made and two small tumour fragments of 6–7 mg were placed under the renal capsule of either kidney, one in the upper pole and one in the lower pole of the kidney. To exclude perfusion defects of the kidneys, the location of inoculation of CC531 and ROS-1 was varied between the upper or lower pole of the kidney. After 7 days, the animals were used for the experiments. At 14 days after inoculation, the animals were sacrificed, the tumours were enucleated and weighed. The tumours in the right kidney were used as an internal control. Nine replicate experiments were performed.

Statistical significance was assessed using the Mann–Whitney *U*-test.

### RESULTS

### Unilateral nephrectomy

Unilateral nephrectomy was very well sustained. There was a minor increase in creatinine shortly after operation, but all animals showed a quick recovery. Kidney functions fluctuated within a normal range.

### Sham perfusion

In five animals, isolated kidney perfusion with perfusate only was performed (Figure 1). BUN and creatinine levels were elevated for 3 days, but returned to normal within 1 week. Blood levels were followed for 3 weeks, but showed only fluctuation. After perfusion, kidneys showed slight oedema, depending on perfusion pressure. Perfusion pressure greater than 120 mmHg resulted in some oedema, kidneys perfused with less than 120 mmHg showed no oedema; thus, all perfusions with TNF-α were performed with a pressure less than 120 mmHg. At sacrifice, no macroscopic abnormalities were seen. All rats survived the procedure.

### TNF-α perfusion

After perfusion with 1 μg TNF-α, a rise in blood urea nitrogen (BUN) and in creatinine levels was observed during the first 4 days after operation (Figure 1). In spite of this initial toxicity, all rats survived the procedure and kidney functions returned to normal after 6 days and remained within normal range. Animals recovered their preoperative weight after a median of 25 days after isolated kidney perfusion. Perfusion with 2 μg of TNF-α resulted in a continuous rise of BUN and creatinine (Figure 1), and rats were sacrificed in bad clinical condition. After 2 days, rats were lethargic and had bloody diarrhoea. After isolated kidney perfusions with 5 μg added to the perfusion circuit, rats died very quickly because of shock and respiratory failure within 24 h (data not shown).

© Cancer Research Campaign 1999

### Histology

Kidneys perfused with perfusate only did not show any major changes in histology (Figure 2A).

In kidneys perfused with 1 μg TNF-α, no severe abnormalities could be seen, but signs of focal tubular necrosis and bleeding were seen in the 2 μg (Figure 2B) and 5 μg groups. Scattered mononuclear inflammatory cells are present in the interstitium. The glomeruli appeared to be relatively unaffected.

### Animal weight

All animals showed a decrease in weight after the perfusion. During the first week, weight gain was minimal, but 21 days after isolated kidney perfusion the animals recovered their preoperative weight.

### In vitro sensitivity of the colon adenocarcinoma CC531 to TNF-α and melphalan

Cells of the CC531 tumour showed only a minor response to increasing dosages of rhTNF-α as determined by the sulpho-rhodamine-B assay. The $IC_{50}$ of cells treated with more than 10 μg $ml^{-1}$ TNF-α was just reached, which means a significant reduction in the number of tumour cells after 48 h of incubation ($P < 0.05$).

The dose–response curve of CC531 cells to melphalan alone (0 μg $ml^{-1}$ TNF-α) showed sensitivity in vitro at dosages higher than 1 μg $ml^{-1}$ (Figure 3A). The $IC_{50}$ of melphalan is reached with a concentration of > 10 μg $ml^{-1}$. The cell line proved to be relatively resistant to the cytotoxic effects of melphalan.

The $IC_{50}$ of the adenocarcinoma cells to treatment with melphalan was only slightly reduced in the presence of incrementing dosages of TNF-α (Figure 3A, for clarity only 0.5 and 10 μg TNF-α is shown). The maximal growth of CC531 is reduced in the presence of TNF-α. Because the dose–response curves all bend towards total growth inhibition, irrespective of the concentration of TNF-α, a synergy between the cytokine and the cytotoxic drug in this tumour system in vitro could not be demonstrated.

### In vitro sensitivity of the osteosarcoma ROS-1 to TNF-α and melphalan

The dose–response curves of the ROS-1 osteosarcoma cell line to TNF-α and melphalan are shown in Figure 3B. The osteosarcoma cell line shows relative minor sensitivity to TNF-α alone (Manusama et al, 1996*a*). The $IC_{50}$ for melphalan is reached at 6 μg $ml^{-1}$.

Maximum growth of the osteosarcoma in vitro at lower dosages of melphalan is reduced in the presence of TNF-α at the various concentrations used (for clarity only 0.5 and 10 μg $ml^{-1}$ TNF-α curves are shown). Total growth inhibition is reached with increasing dosages of melphalan, almost independent of TNF-α. These experiments, therefore, could not reveal synergism of TNF-α and melphalan in the tumour cytotoxic effects, but an additive effect at best.

### Tumour response of CC531

In the SRCA, the relatively low concentration of 0.2 μg $ml^{-1}$ TNF-α had to be used. Kidney functions were severely disturbed at higher dosages. Sham perfusion and isolated kidney perfusion with 0.2 μg $ml^{-1}$ TNF-α under oxygenated conditions showed no significant inhibition of tumour growth (data not shown). There was minor growth inhibition of the solid tumour in this location, but none of the tumours showed regression.

A combination of the MTD of TNF-α (1 μg) and the dose used in isolated limb perfusions of 40 μg melphalan was chosen at first to investigate whether there were any synergistic or additive effects in vivo. We could not prove significant growth inhibition with this combination.

In subsequent experiments, we tested the combination of a high-dose melphalan (500 μg) with the MTD of TNF-α (1 μg). These experiments showed growth inhibition, but no significance was reached (Figure 4, $n = 9$; treated vs control; mean 57 mg vs 81.11 mg, s.d. 12.59 vs 13.22, $P = 0.1999$).

### Tumour response of osteosarcoma

In this tumour model, only minor sensitivity was shown to the maximally tolerated dosages of TNF-α and melphalan. Mean tumour weight ($n = 9$; treated 76.0 ± 11.91 mg vs control 105.8 ± 12.76 mg; $P = 0.157$) was only slightly reduced, again without significance (Figure 4).

### DISCUSSION

Isolated perfusion with tumour necrosis factor-α in combination with ischaemia (Manusama et al, 1994) or with melphalan (Manusama et al, 1996*a*, 1996*b*) has been reported to result in complete tumour regression in preclinical tumour models. Isolated limb perfusion in patients with in-transit melanoma with TNF-α in combination with melphalan and γ-interferon resulted in high complete remission rates (Liénard et al, 1992, 1994). Also, high limb salvage rates have been reported with the same treatment protocol in patients with non-resectable soft-tissue sarcomas (Eggermont et al, 1993, 1996*a*, 1996*b*).

Isolated organ perfusion with TNF-α is clearly a more complicated matter than isolated limb perfusion. Different models have been developed to evaluate the efficacy of TNF-α in organ perfusion, such as in lung (Weksler et al, 1993, 1994; Pogrebniak et al, 1994), and in liver (Fraker et al, 1994; Borel Rinkes et al, 1997). TNF-α in isolated lung perfusion has been shown to be safe, and in a phase I study tumour responses have been observed in patients with lung metastases (Pass et al, 1996).

The isolated perfused kidney model has been used to study the effects of cytotoxic agents (Asbach and Bersch, 1980). Here, we demonstrate that analogous to the isolated limb perfusion (ILP) isolated perfusion of the kidney (IKP) is technically feasible.

In the current model, the MTD was reached at 1 μg of TNF-α, showing only a transient renal toxicity. At 2 μg of TNF-α, fatal renal toxicity was seen. This involved acute renal failure leading to death by day 4. An acute fatal shock syndrome was noted at 5 μg.

It is known that TNF-α may have direct toxic effects to the kidney (Tracey et al, 1986; Gaskill, 1988; Kahky et al, 1990). Acute tubular necrosis was seen with portal infusion of sublethal doses of TNF-α with relative sparing of the glomeruli. In surviving animals, a decrease in kidney function was noted. The serious toxicity seen in our experiments with higher dosages of TNF-α may be partially explained by the production of TNF-α by glomerular macrophages, mesangial cells and renal tubular cells upon stimulation (Affres et al, 1991; Tipping et al, 1991; Baud and Ardaillou, 1995). Analogous to the production of TNF-α and interleukin 1 by Kupffer cells (Kahky et al, 1990), we hypothesize

© Cancer Research Campaign 1999

that a potent second cytokine release is responsible for the increased toxicity. In rats perfused with 5 μg, the secondary cytokine response was so extreme that the rats die of acute respiratory distress. Similar observations have been reported by Fraker et al (1994) in pigs, shown to be due to a secondary cytokine response that could not be prevented by anti-TNF-α antibody treatment. Toxicity to the lung is manifested by pulmonary oedema and adult respiratory distress syndrome (Pogrebniak et al, 1994). Thus, in contrast to the very high concentration of TNF-α that can be applied in the ILP setting, the kidney proves to be a very susceptible organ which only tolerates 1/50th of the TNF-α dose used in ILP.

In vitro, synergy between TNF-α and melphalan for the rat colon adenocarcinoma cell line CC531 was not observed. Also, synergy could not be proven for the osteosarcoma cell line ROS-1. Here, an additive effect at best is reached.

For in vivo studies, two solid tumour systems were chosen with a different vascularization pattern. The rationale for this choice is based on our previous work with the highly vascularized soft tissue sarcoma BN175, in which the synergistic anti-tumour effects with the combination of TNF-α with melphalan were shown to induce vascular changes accompanied by increased vascular permeability and platelet aggregation (Manusama et al, 1996*a*; Nooijen et al, 1996).

The observations in our in vivo experiments made clear, however, that no strong synergistic anti-tumour effects existed in either tumour. Instead of the high concentration of 10 μg $ml^{-1}$ TNF-α as used in isolated limb perfusion with ROS-1, the relatively low dose of 0.2 μg $ml^{-1}$ had to be used because of dose-limiting toxicity. Thus, the minimal threshold concentration of TNF-α was not reached and, therefore, the crucial vascular effects in vivo described previously (Manusama et al, 1996a; Nooijen et al, 1996) are not observed. Because isolated kidney perfusion in the rat allowed only low dosages of TNF-α, the dual role of TNF-α on the tumour vasculature may be an explanation for the discrepancies between in vitro and in vivo results. It has been demonstrated that low TNF-α concentrations are promoting angiogenesis, whereas high concentrations of TNF-α are toxic to the vessels (Fajardo et al, 1992). The concentration of TNF-α used in isolated kidney perfusion is only 0.2 μg $ml^{-1}$. At this concentration, a promotion of angiogenesis might even be more plausible than the vascular destruction seen with higher dosages. Thus, the typical effects of TNF-α are not seen, which is an explanation for the absence of growth retardation in the tumour models used in our experiments.

Although the model of isolated kidney perfusion was developed to evaluate the effect of TNF-α in isolated organ perfusion, it is also possible to treat kidney tumours with this regimen. Because the main target of TNF-α is the vascular endothelium, well-vascularized renal tumours could potentially be responsive. In a recently published study of isolated perfusion of the kidney in a miniature swine, it appeared possible to perfuse the kidney with 1 mg $ml^{-1}$ (Walther et al, 1996).

We conclude from our studies with the isolated kidney perfusion model that because only 1/50th of the TNF-α concentration was tolerated the advantage of regional application is lost and perspectives for efficacy in tumour-bearing species is much reduced. If the dose needed for anti-tumour effect is 50 times higher than the maximal tolerated dose in isolated kidney perfusion (Asher et al, 1987; Liénard et al, 1992, 1994; Eggermont et al, 1996*a*, 1996*b*), the outlook for clinical applicability seems to be poor.

### ACKNOWLEDGEMENT

This study was made possible by a grant from the Dutch Cancer Association.

### REFERENCES

Affres H, Perez J, Hagege J, Fouqueray B, Kornprobst M, Ardaillou R and Baud L (1991) Desferrioxamine regulates tumor necrosis factor release in mesangial cells. *Kidney Int* **39**: 822–830

Asbach HW and Bersch W (1980) The effect of in situ isolated perfusion of experimental renal tumors with cytotoxic agents in high concentration. *Urol Int* **35**: 112–124

Asher A, Mule JJ, Reichert CM, Shiloni E and Rosenberg SA (1987) Studies on the anti-tumor efficacy of systemically administered recombinant tumor necrosis factor against several murine tumors in vivo. *J Immunol* **138**: 963–974

Baud L and Ardaillou R (1995) Tumor necrosis factor in renal injury. *Miner Electrolyte Metab* **21**: 336–341

Borel Rinkes IHM, de Vries MR, Jonker AM, Swaak TJG, Hack CE, Nooijen PTGA, Wiggers T and Eggermont AMM (1997) Isolated hepatic perfusion in the pig with TNF-α with and without melphalan. *Br J Cancer* **75**: 1447–1453

Brouckaert PGG, Leroux-Rouls GG, Guisez Y, Tavernier J and Fiers W (1986) In vivo anti tumor activity of recombinant human and murine TNF, alone and in combination with murine IFN-gamma on a syngeneic murine melanoma. *Int J Cancer* **38**: 763–769

Dealtry GB, Naylor MS, Fiers W and Balkwill FR (1987) The effect of recombinant human tumor necrosis factor on growth and macromolecular synthesis of human epithelial cells. *Exp Cell Res* **170**: 428–438

Eggermont AMM, Liénard D, Schrafford Koops H, Rosenkaimer F and Lejeune FJ (1993) Treatment of irresectable soft tissue sarcomas of the limbs by isolation perfusion with high dose TNFα in combination with interferon-gamma and melphalan. In *Tumour Necrosis Factor: Molecular and Cellular Biology and Clinical Relevance*. Fiers W and Buurman WA (eds.), pp. 239–243. Karger: Basle

Eggermont AMM, Schraffordt Koops H, Liénard D, Kroon BBR, van Geel AN, Hoekstra HJ and Lejeune FJ (1996*a*) Isolated limb perfusion with high dose tumor necrosis factor-α in combination with Interferon-γ and melphalan for nonresectable extremity soft tissue sarcomas: a multicenter trial. *J Clin Oncol* **14**: 2653–2665

Eggermont AMM, Schraffordt Koops H, Klausner J, Kroon BBR, Schlag PM, Liénard D, Van Geel AN, Hoekstra HJ, Meller I, Nieweg OE, Kettelhack C, Ben-Ari G, Pector JC and Lejeune FJ (1996*b*) Isolated limb perfusion with tumor necrosis factor and melphalan for limb salvage in 186 patients with locally advanced extremity sarcomas. The cumulative multicenter European experience. *Ann Surg* **224**: 756–765

Fajardo LF, Kwan HH, Kowalski J, Prionas SD and Allison AC (1992) Dual role of tumor necrosis factor-α in angiogenesis. *Am J Pathol* **140**: 539–544

Feinberg B, Kurzrock R, Talpaz M, Blick M, Saks S and Gutterman JU (1988) A phase I trial of intravenously-administered recombinant tumor necrosis factor-alpha in cancer patients. *J Clin Oncol* **6**: 1328–1334

Fine LG (1986) The biology of renal hypertrophy. *Kidney Int* **29**: 619–634

Fraker DL, Alexander HR and Thom AK (1994) Use of tumor necrosis factor in isolated hepatic perfusion. *Circulatory Shock* **44**: 45–50

Gaskill HV (1988) Continuous infusion of tumor necrosis factor: mechanisms of toxicity in the rat. *J Surg Res* **44**: 664–671

Hieber U and Heim ME (1994) Tumor necrosis factor for the treatment of malignancies. *Oncology* **51**: 142–153

Kahky MP, Daniel CO, Cruz AB and Gaskill HV (1990) Portal infusion of tumor necrosis factor increases mortality in rats. *J Surg Res* **49**: 138–145

Kallinowski F, Schaeffer C, Tyler G and Vaupel P (1989) In vivo targets of recombinant human tumor necrosis factor-α: blood flow, oxygen consumption and growth of isotransplanted art tumors. *Br J Cancer* **60**: 555–560

Kramer SM and Carver ME (1986) Serum-free in vivo bioassay for the detection of tumor necrosis factor. *J Immunol Methods* **93**: 201–206

Lejeune FJ, Liénard D, Leyvraz S and Mirimanoff RO (1993) Regional therapy of melanoma. *Eur J Cancer* **29A**: 606–612

Liénard D, Ewalenko P, Delmotte JJ, Renard N and Lejeune FJ (1992) High-dose recombinant tumor necrosis factor alpha in combination with interferon gamma and melphalan in isolation perfusion of the limbs for melanoma and sarcoma. *J Clin Oncol* **10**: 52–60

Liénard D, Eggermont AMM, Schraffordt Koops H, Kroon BB, Rosenkaimer F, Autier P and Lejeune FJ (1994) Isolated perfusion of the limb with high-dose

 *© Cancer Research Campaign 1999*

tumor necrosis factor-alpha (TNF-alpha), interferon-gamma (IFN-gamma) and melphalan for melanoma stage III. Results of a multi-centre pilot study. *Melanoma Res* **4**: 21–26

Maessen JG, Greve JWM and Buurman WA (1991) Increased sensitivity to endotoxemia by tissue necrosis. *Surgery* **109**: 154–159

Manusama ER, Marquet RL, Durante NMC and Eggermont AMM (1994) Ischemia promotes the antitumour effect of tumor necrosis factor-α (TNFα) in isolated limb perfusion in the rat. *Reg Cancer Treat* **7**: 155–159

Manusama ER, Nooijen PTGA, Stavast J, Durante NMC, Marquet RL and Eggermont AMM (1996*a*) Synergistic antitumor effect of recombinant human tumor necrosis factor α with melphalan in isolated limb perfusion in the rat. *Br J Surg* **83**: 551–555

Manusama ER, Stavast J, Durante NMC, Marquet RL and Eggermont AMM (1996*b*) Isolated limb perfusion with TNFα and melphalan in a rat osteosarcoma model: a new anti-tumor approach. *Eur J Surg Oncol* **22**: 152–157

Marquet RL, Westbroek DL and Jeekel J (1984) Interferon treatment of a transplantable rat colon adenocarcinoma: importance of tumor site. *Int J Cancer* **33**: 689–692

Nooijen PTGA, Manusama ER, Schalkwijk L, de Waal RMW, Eggermont AMM and Ruiter DJ (1996) Histopathologic analysis of the synergistic antitumor effects of TNFα and melphalan in an isolated limb perfusion model of rat sarcoma. *Br J Cancer* **74**: 1908–1915

Pass HI, Mew DJY, Kranda KC, Temeck BK, Donington JS and Rosenberg SA (1996) Isolated lung perfusion with tumor necrosis factor for pulmonary metastases. *Ann Thorac Surg* **61**: 1609–1617

Pogrebniak HW, Witt CJ, Terrill R, Kranda K, Travis WD, Rosenberg SA and Pass HI (1994) Isolated lung perfusion with tumor necrosis factor: a swine model in preparation of human trials. *Ann Thorac Surg* **57**: 1477–1483

Prehn RT and Main JW (1957) Immunity to methylcholantrene-induced sarcomas. *J Natl Cancer Inst* **18**: 769–778

Shimomura K, Manda T, Mukumoto S, Kobayashi K, Nakano K and Mori J (1988) Recombinant human tumor necrosis factor-α: thrombus formation is a cause of anti-tumour activity. *Int J Cancer* **41**: 243–247

Sidhu RS and Bollon AP (1993) Tumor necrosis factor activities and cancer therapy – a perspective. *Pharm Ther* **57**: 79–128

Skehan P, Storeng R, Scudiero D, Monks A, McMahon J, Vistica D, Warren JT, Bokesch H, Kenney S and Boyd MR (1990) New colorimetric cytotoxicity assay for anticancer-drug screening. *J Natl Cancer Inst* **82**: 1107–1112

Spriggs DR and Yates SW (1992) Cancer chemotherapy. Experience with TNF administration in humans. In *Tumor Necrosis Factors: The Molecules and their Emerging Role in Medicine*. Beutler B (ed.), pp. 383–406. Raven Press: New York

Spriggs DR, Sherman ML, Michie H, Arthur KA, Imamura K, Willmore D, Frei 3rd E and Kufe DW (1988) Recombinant human tumor necrosis factor administered as a 24 h intravenous infusion. A phase I and pharmacologic study. *J Natl Cancer Inst* **80**: 1039–1044

Tipping PG, Leong TW and Holdsworth SR (1991) Tumor necrosis factor production by glomerular macrophages in anti-glomerular basement membrane glomerulonephritis in rabbits. *Lab Invest* **65**: 272–279

Tracey KJ, Beutler B, Lowry SF, Merryweather J, Wolpe S, Milsark IW, Hariri RJ, Fahey TJ, Zentella A, Albert JD, Shires GT and Cerami A (1986) Shock and tissue injury induced by recombinant human cachectin. *Science* **234**: 470–474

Walther MM, Jennings SB, Choyke PL, Andrich M, Hurley K, Marston Linehan W, Rosenberg SA and Alexander RB (1996) Isolated perfusion of the kidney with tumor necrosis factor for localized renal-cell carcinoma. *World J Urol* **14**: S2–S7

Watanabe N, Niitsu Y, Umeno H, Sone H, Neda H, Yamauchi N, Maeda M and Urushizaki I (1988) Toxic effect of TNF on tumor vasculature in mice. *Cancer Res* **49**: 2179–2183

Weksler B, Schneider A, Ng B and Burt ME (1993) Isolated single lung perfusion in the rat: an experimental model. *J Appl Physiol* **74**: 2736–2739

Weksler B, Blumberg D, Lenert JT, Ng B, Fong Y and Burt ME (1994) Isolated single-lung perfusion with TNF-α in a rat sarcoma lung metastasis model. *Ann Thorac Surg* **58**: 328–332

© Cancer Research Campaign 1999
